# Tip-in underwater endoscopic mucosal resection for a flat gastric tumor

**DOI:** 10.1055/a-2719-8448

**Published:** 2025-11-05

**Authors:** Kazuki Matsuyama, Minoru Kato, Shunsuke Yoshii, Tomoki Michida, Ryu Ishihara

**Affiliations:** 1 53312 Department of Gastrointestinal Oncology, Osaka International Cancer Institute, Osaka, Japan

Underwater endoscopic mucosal resection (UEMR) has been reported as a simple method for resecting small gastric lesions 1 2 . However, due to the thickness of the gastric wall, slippage of the snare tip occasionally occurs, especially with flat or depressed lesions. Tip-in UEMR is a new technique recently reported for duodenal and colorectal lesions 3 4 , which enables secure anchoring of the snare tip by making the mucosal incision at the distal side of the lesion. Herein, we report the first case of a gastric flat tumor treated with tip-in UEMR ( Video 1 ).

Tip-in underwater endoscopic mucosal resection for a flat gastric tumor.Video 1

A woman in her 30s with familial adenomatous polyposis underwent endoscopy, which revealed an 11-mm flat lesion on the greater curvature of the lower gastric body ( Fig. 1 ). The biopsy result was adenoma. As the lesion was a small adenoma, endoscopic submucosal dissection was deemed both time-consuming and overtreatment; therefore, snare resection was chosen as a treatment approach. Given the flat nature of the lesion, conventional UEMR was anticipated to cause snare tip slippage. We thus attempted a tip-in UEMR.

**Fig. 1:**
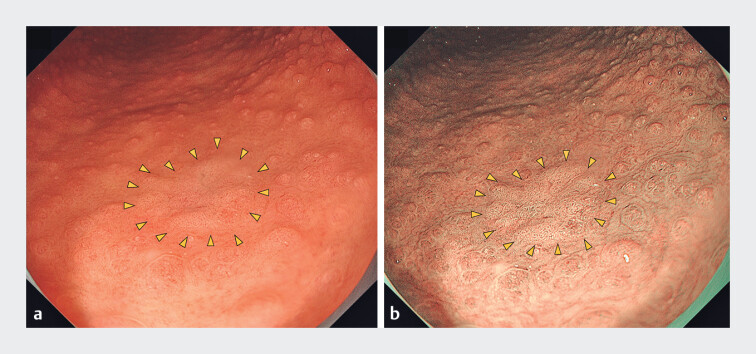
During follow-up endoscopy in a patient with familial adenomatous polyposis, an 11-mm, whitish flat lesion (yellow arrowheads) was identified on the greater curvature of the lower gastric body. **a** White light imaging. **b** Narrow band imaging.

After marking around the lesion with snare tip (SnareMaster Plus [15 mm]; Olympus, Tokyo, Japan) ( Fig. 2 **a** ), underwater condition was established, and 1 mL of saline was injected into the normal mucosa at the anal side of the lesion ( Fig. 2 **b** ). Mucosal incision was then performed using the tip of the snare ( Fig. 2 **c** ). After inserting and fixing the snare tip into the submucosa, the snare was gradually expanded to include the entire markings ( Fig. 2 **d** ) and subsequently closed for *en bloc* resection. The ulcer base was then cauterized ( Fig. 3 ) and clipped. No adverse events were observed. The time from water infusion to resection was 4 minutes. Histology revealed an adenoma with negative horizontal and vertical margins.

**Fig. 2:**
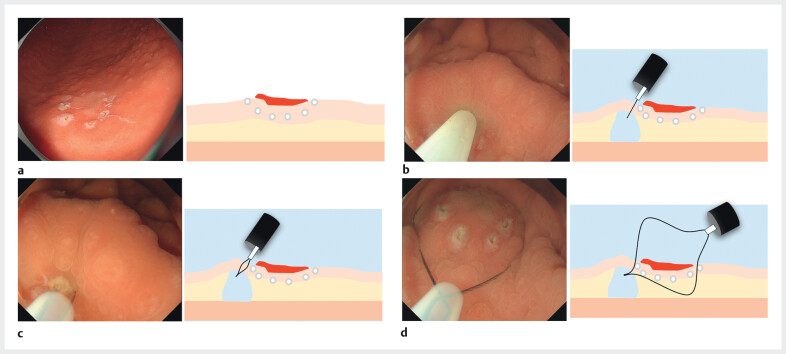
Schema and images of the technique used in this case. **a** First, marking was performed around the lesion using the tip of the snare. **b** Underwater conditions, 1 mL of saline was injected into the normal mucosa at the anal side of the lesion. **c** An incision was made in the elevated portion using the snare, and the snare tip was fixed (tip-in). **d** The snare was carefully closed, ensuring that all markings were included within it.

**Fig. 3:**
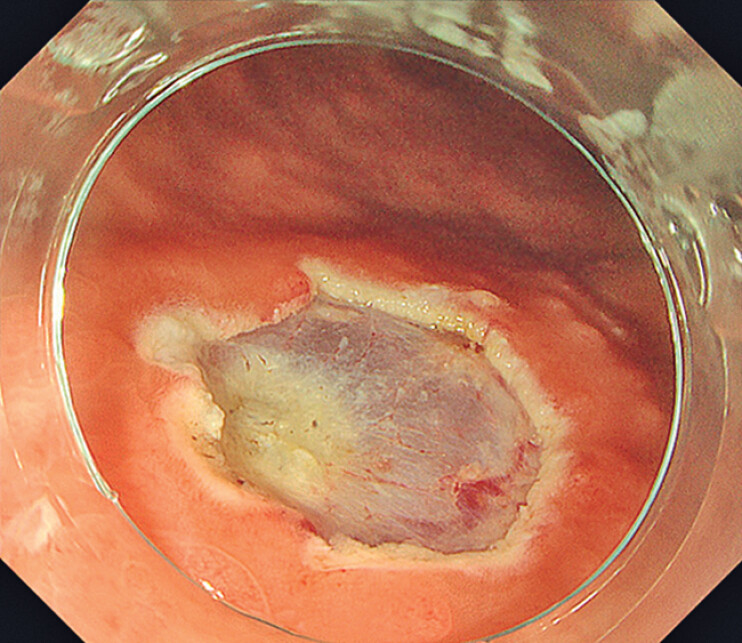
Post-resection ulcer after tip-in underwater endoscopic mucosal resection showing complete lesion removal.

Tip-in UEMR, a simple technique requiring no special skills, would be a useful option for resecting small gastric tumors.

Endoscopy_UCTN_Code_TTT_1AO_2AG_3AB

## Notes

Endoscopy E-Videos https://eref.thieme.de/e-videos
*E-Videos* is an open access online section of the journal *Endoscopy* , reporting on interesting cases and new techniques in gastroenterological endoscopy. All papers include a high-quality video and are published with a Creative Commons CC-BY license. Endoscopy E-Videos qualify for HINARI discounts and waivers and eligibility is automatically checked during the submission process. We grant 100% waivers to articles whose corresponding authors are based in Group A countries and 50% waivers to those who are based in Group B countries as classified by Research4Life (see: https://www.research4life.org/access/eligibility/ ). This section has its own submission website at https://mc.manuscriptcentral.com/e-videos .
